# Gut Mycobiome in Atopic Dermatitis and in Overweight Young Children: A Prospective Cohort Study in Finland

**DOI:** 10.3390/jof10050333

**Published:** 2024-05-04

**Authors:** Petri Vänni, Jenni Turunen, Ville K. Äijälä, Vilja V. Tapiainen, Marika Paalanne, Tytti Pokka, Niko Paalanne, Mysore V. Tejesvi, Terhi S. Ruuska

**Affiliations:** 1 Research Unit of Clinical Medicine, University of Oulu, 8000 Oulu, Finland; jenni.turunen@oulu.fi (J.T.); marika.paalanne@oulu.fi (M.P.); tytti.pokka@oulu.fi (T.P.); niko.paalanne@oulu.fi (N.P.); mysore.tejesvi@oulu.fi (M.V.T.); terhi.ruuska@oulu.fi (T.S.R.); 2 Biocenter Oulu, University of Oulu, 8000 Oulu, Finland; 3 Department of Paediatrics and Adolescent Medicine, Oulu University Hospital, 8000 Oulu, Finland; ville.aijala@student.oulu.fi (V.K.Ä.); vilja.tapiainen@student.oulu.fi (V.V.T.); 4 Research Service Unit, Oulu University Hospital, 8000 Oulu, Finland; 5 Ecology and Genetics, Faculty of Science, University of Oulu, 8000 Oulu, Finland

**Keywords:** infants, children, obesity, atopic dermatitis, atopy, microbiome, mycobiome, fungi, machine learning, prospective

## Abstract

Gut bacterial alterations have been previously linked to several non-communicable diseases in adults, while the association of mycobiome is not well understood in these diseases, especially in infants and children. Few studies have been conducted on the association between gut mycobiome and non-communicable diseases in children. We investigated gut mycobiome composition using 194 faecal samples collected at birth, 6 months after birth, and 18 months after birth in relation to atopic dermatitis (AD) and overweight diagnoses at the age of 18 or 36 months. The mycobiome exhibited distinct patterns, with *Truncatella* prevalent in the meconium samples of both overweight and non-overweight groups. *Saccharomyces* took precedence in overweight cases at 6 and 18 months, while *Malassezia* dominated non-overweight samples at 6 months. *Saccharomyces* emerged as a consistent high-abundance taxon across groups that had dermatitis and were overweight. We found a weak association between gut mycobiome and AD at birth and overweight at 18 months when using machine learning (ML) analyses. In ML, unidentified fungi, *Alternaria*, *Rhodotorula,* and *Saccharomyces*, were important for classifying AD, while *Saccharomyces*, *Thelebolus,* and *Dothideomycetes* were important for classifying overweight. Gut mycobiome might be associated with the development of AD and overweight in children.

## 1. Introduction

The human gut contains a vast number of microorganisms, including bacteria, viruses, archaea, and fungi. This diverse ecosystem plays a crucial role in regulating various bodily functions, such as metabolism [1], immune system function [2], and the communication between the gut and brain [3]. The gut bacterial microbiome has been previously linked to non-communicable diseases (NCDs), such as AD [4,5,6], coronary artery disease [7], inflammatory bowel disease [8], type 2 diabetes [9], gastrointestinal cancer [10], and obesity [11]. AD is a prevalent NCD among Finnish children, with approximately 25% of children in Finland experiencing AD during early life [12]. Overweight, including obesity, is also common in Finland, affecting 18% to 27% of Finnish children [13].

Most previous studies have focused on the gut bacteriome, while the role of fungi, also known as the mycobiome, is still largely unknown. Gut mycobiome exhibits significant variability within and between individuals [14,15]. Understanding the composition and function of gut mycobiome is crucial for further elucidating its roles in human health and developing potential therapies for microbiome-related disorders. Adult gut mycobiome alterations have been previously linked to NCDs [16], such as inflammatory bowel disease [17,18], obesity [19], autism [20], and several cancers [21,22]. Only a few studies, mostly using cross-sectional study designs, have been conducted on the association of NCDs and the gut mycobiome in children, mainly in relation to inflammatory bowel disease [23,24,25,26,27,28,29]. The relationship between the mycobiome in early life and the subsequent diagnosis of AD or overweight has not been comprehensively investigated, although specific bacterial taxa have been associated with an increased risk of AD in children later on [6].

In the present study, we set out to investigate the association of gut mycobiome with atopic dermatitis and overweight in young children in a prospective cohort followed up from birth until the age of 3 years.

## 2. Materials and Methods

### 2.1. Study Design and Population

This prospective cohort study consisted originally of 508 newborn infants born at Oulu University Hospital, Finland, between April 2016 and December 2018. The infants were assessed with electronic questionnaires at the ages of 18 and 36 months. Maturation of mycobiome was assessed by faecal samples at birth and at the ages of 6 and 18 months. Children were categorised as having AD if their parents initially reported AD in any of the follow-up questionnaires, which was subsequently confirmed by a physician’s diagnosis. Bioinformatics was performed for 194 samples that had the necessary questionnaire data for analyses.

Growth data were systematically collected from electronic medical records of the child welfare clinics, where trained nurses measured the length and weight of the children using standardised techniques. In Finland, weight in children under 2 years of age is assessed as a percentage of the weight-for-length/height in child welfare clinics. Children under 7 years of age with a percentage of the weight-for-length/height ≥ 10% are considered to be overweight and with ≥20% are considered obese according to national guidelines intended as a reference for monitoring growth in Finnish population [30].

The study samples were initially categorised based on the timing of faecal mycobiota sampling: post-birth, 6 months after birth, and 18 months after birth. Within each of these time points, the samples were further divided into four groups: individuals diagnosed with AD or overweight after faecal mycobiota sampling at either 18 or 36 months of age, and those without such diagnoses. In these groups some samples could be independently counted as two different groups. Additionally, the samples were partitioned into a new set of non-overlapping groups: individuals with AD who were of normal weight, individuals who were overweight but not diagnosed with AD, individuals diagnosed with both AD and overweight, and individuals without AD who were of normal weight. The study protocol was evaluated and approved by the ethical committee of the Northern Ostrobothnia Hospital District at Oulu University Hospital, Oulu, Finland (Decision 3/2016). The families provided written informed consent in advance, and the study was conducted in accordance with applicable regulations and standards.

### 2.2. DNA Extraction

DNA was extracted using the DNeasy PowerSoil Pro kit from Qiagen, Hilden, Germany. Extraction was conducted using the protocol provided by the manufacturer. To begin, 1 mL of phosphate-buffered saline was added per sample, and samples were homogenised with bead beating. However, samples with low biomass were homogenised using a vortex adapter protocol. Extraction was then performed using a QIAcube Connect machine from Qiagen. The quality of DNA was subsequently quantified with spectrophotometry (NanoDrop, Thermo Fisher Scientific, Waltham, MA, USA).

### 2.3. DNA Sequencing

The ITS2 gene was sequenced using the fITS7b (5′-GTGARTCATCGAATCTTTG-3′) and ITS4 (5′-TCCTCCGCTTATTGATATGC-3′) primers. Before sequencing, PCR was conducted utilizing the Phusion Flash High-Fidelity PCR master mix from Thermo Fisher Scientific. The following PCR protocol was followed: 2 min of initialisation, 35 repetitions of denaturation at 98 °C for 10 s, annealing at 54 °C for 20 s, and elongation at 72 °C for 30 s, with the final elongation lasting for 7 min. Amplicon sequencing was executed using an IonTorrent PGM platform. Sequencing was performed in four separate runs. Details about DNA extraction, PCR, and sequencing have been previously published [31].

### 2.4. Sequence Preprocessing

The mycobiome sequences and metadata of each sample were imported into Qiime2 v2022.8 using the tools plugin [32]. Sequencing primers were removed using a cutadapt plugin in Qiime2 with default settings, with the only deviation being a 0.1 error rate parameter setting. Samples were denoised into amplicon sequence variants (ASVs) with the DADA2 plugin, trimming the first 15 base pairs and truncating to a length of 160, producing a feature table and representative sequence files. Quality filtering steps were avoided before DADA2, as DADA2 includes native quality and chimera filtering. Each sequencing run was processed independently up to denoising, and afterwards, combined into a single feature table and representative sequence files using the feature-table plugin in Qiime2. Chimeric sequences were further removed using a vsearch [33] plugin with uchime-denovo command with default parameters in Qiime2. Contaminations were removed using the R-package Decontam [34] with a prevalence threshold of 50%. Then, samples with fewer than 500 total reads were excluded to filter out samples with a low number of high-quality sequences using the feature-table plugin and filter-samples command in Qiime2. Extremely rare features, which were found in less than two samples and with a total frequency of 10 or less, were removed with the feature-table plugin. The naïve Bayes taxonomic classifier was trained using Qiime2 with the feature-classifier plugin and classify-sklearn command with default parameters using the full-length UNITE [35] v16.10 database. All features classified as mitochondria or chloroplasts were removed with the taxa plugin in Qiime2. Additionally, all features classified as non-fungal were removed. Afterwards, two feature tables were generated, one in which all features were binned to the genera level or to the next closest taxonomic level available, and one in which taxonomic labels were not assigned, and features were kept as ASVs instead. We had 194 samples for the analysis (Table 1).

### 2.5. Alpha and Beta Diversity

The alpha and beta diversity were computed using the diversity plugin in Qiime2. Alpha diversity was evaluated using the Shannon index, while beta diversity was assessed using the Bray–Curtis dissimilarity. Beta diversity, between sample diversity, was transformed and visualised with principal coordinate analysis, where confidence ellipses were drawn using the Pearson correlation coefficient for each group. Before the diversity analyses, the feature tables were rarefied to control for uneven sequencing depth, while non-rarefied data were used for other analyses [36]. Rarefication depth was selected so that the minimum depth was 500, and the next highest depth without loss of samples was selected for each subgroup. A rarefication depth of 504 was selected for meconium, 563 for 6 months, and 1259 for 18 months. Kruskal–Wallis H tests were used to compare differences in alpha diversity between the study groups using the Scipy python package with a chosen significance threshold of 0.05. Meanwhile, the Adonis (2.5-3) software Qiime2 was utilised to investigate differences between groups in beta diversity using the transformed principal coordinate analysis of transformed data using PERMANOVA. The Benjamini–Hochberg procedure, as implemented in the statsmodels python package, was used for multiple testing correction in both alpha and beta diversity analyses. All *p*-values from statistical tests that were lower than 0.05 were considered significant.

### 2.6. Differential Abundance Analysis

The differences in feature abundance between study groups were assessed using the “analysis of composition of microbiomes II” (ANCOM-II) [37] method in the R programming environment. ANCOM-II can be employed to compare the microbiome composition in two or more groups and identify microbes that are more or less abundant in one group relative to another. Prior to ANCOM-II analyses, rare features—defined as those found in less than 10% of the total samples—were removed from the feature table. The statistical tests between each feature abundance were performed in a pairwise manner using ANCOM-II. A non-repeated and non-adjusted analysis pipeline was selected in accordance with the documentation, utilizing the Kruskal–Wallis H test for pairwise comparisons and the Benjamini–Hochberg procedure for multiple adjustments. According to the ANCOM-II documentation, a feature is generally considered significant if 70% or more of the pairwise statistical tests for that feature are inferred as statistically significant. However, to mitigate the false discovery rate associated with multiple target variables, a significance threshold was chosen such that a feature was deemed differentially abundant only if more than 90% of all pairwise tests passed.

### 2.7. Machine Learning Analysis

We used random forest [38] machine learning (ML) models to predict the subsequent NCDs using data on gut mycobiome in the faecal samples collected during infancy. Random forest is a supervised ML algorithm that functions as an ensemble of weak learners, culminating in a robust predictor based on the collective input of these learners. The individual weak learners in random forest models are decision trees. Due to limited sample sizes, no parameter tuning or feature selection methods were included in the model building pipeline. The parameters of the random forest models were pre-selected before analysis. Each model comprised 250 decision trees, utilised Gini impurity to measure the quality of splits and considered the square of the number of total features in the data as the maximum features for each split. Each sample was assigned weights inversely proportional to class frequencies to control the unbalanced nature of the dataset during model training. All other parameters were set as the default option. Random forest models generate an internal metric of feature importance during training, calculating the mean reduction in Gini impurity when a feature is selected for splitting. Models were trained on both ASVs and genera collapsed feature tables independently. A leave-one-out cross-validation (LOOCV) method was employed to evaluate the performance of the ML models, where one sample at a time was excluded from the training fold and tested with the model built on all other samples. LOOCV was chosen to maximise the amount of training data in each iteration because the sample size was low in some study groups. Model performances were evaluated using a Receiver Operating Characteristic (ROC) curve and a Precision–Recall (PR) curve. In this study, ROC Area Under the Curve (AUC) values were interpreted as follows: 0.4–0.6 as close to random chance, 0.6–0.7 as poor, 0.7–0.8 as mediocre, 0.8–0.9 as good, and 0.9–1.0 as excellent. LOOCV runs were repeated 40 times, and ROC curves were averaged across repeats to estimate performance. Similarly, feature importance, measured as mean decrease impurity (MDI), was averaged across repeated LOOCV runs. To generate PR curves, predictions were pooled across repetitions, and a single PR curve was generated from the test predictions of all repetitions. The ML analyses were performed using the Scikit-learn v1.1.2 [39] Python package and visualised with Matplotlib [40].

## 3. Results

### 3.1. Descriptive Statistics of Study Samples

At birth, there were 98 fungal taxa and 254 ASVs in the first-pass meconium, whereas 30 taxa and 61 ASVs were found in faecal samples obtained at 6 months of age and 46 taxa and 118 ASVs at the age of 18 months. Clinical characteristics of individuals in each study group are presented in the Supplementary Table S1.

**Figure 1:**
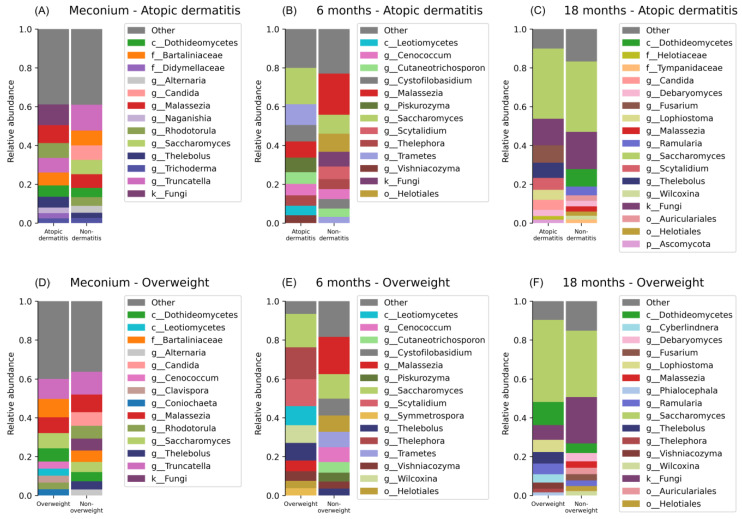
Relative abundance of main fungal genera in faecal samples according to subsequent clinical outcomes. Faecal samples were collected at birth, 6 months, and 18 months of age. Clinical outcomes were AD (**A**–**C**) or overweight (**D**–**F**) reported by parents at the age of 18 months, 3 years, or both. The 10 most abundant taxa are shown. Before each name, the taxonomic level is described as a single letter such as kingdom (k), phylum (p), class (c), family (f), and genus (g). Taxa were collapsed to the last classified level down to genus.

The highest average abundance taxa varied between the AD and non-dermatitis groups at birth and 6 months (Figure 1, Supplementary Table S2). In the meconium non-dermatitis group, *Truncatella* (mean = 0.13, SD = 0.21) was the most abundant genus, while unidentified fungal genera (mean = 0.1, SD = 0.27) were the most abundant in the AD group. At 6 months of age, *Malassezia* (mean = 0.21, SD = 0.37) and *Saccharomyces* (mean = 0.1, SD = 0.22) were the most abundant taxa in the non-dermatitis samples, while *Saccharomyces* (mean = 0.19, SD = 0.25) and *Trametes* (mean = 0.11, SD = 0.26) were the highest abundant in the AD cases. At 18 months of age, *Saccharomyces* (AD: mean = 0.36, SD = 0.29, non-AD: mean = 0.36, SD 0.38) and unidentified fungi (AD: mean = 0.14, SD = 0.26, non-AD: mean = 0.19, SD 0.32) dominated the first and second highest abundance positions in the AD and non-dermatitis groups (Figure 1, Supplementary Table S2).

The highest relative abundance taxa in the meconium samples were *Truncatella* in both the overweight (mean = 0.1, SD = 0.21) and non-overweight (mean = 0.12, SD = 0.2) groups, while the second highest was *Bartaliniaceae* (mean = 0.09, SD = 0.21) in the overweight group and *Malassezia* (mean = 0.9, SD = 0.22) in the non-overweight group (Figure 1, Supplementary Table S2). At 6 months of age, in the overweight group, *Saccharomyces* was the most abundant taxon (mean = 0.17, SD = 0.27), followed by *Thelepora* as the second most abundant taxon (mean = 0.16, SD = 0.33). In the non-overweight group, *Malassezia* emerged as the most abundant taxon (mean = 0.19, SD = 0.36), while *Saccharomyces* ranked as the second highest (mean = 0.13, SD = 0.36). For samples collected at 18 months, *Saccharomyces* (mean = 0.42, SD = 0.33) and *Dothideomycetes* (mean = 0.12, SD = 0.28) were the two highest abundance taxa in the overweight group, while *Saccharomyces* (mean = 0.34, SD = 0.38) and unidentified fungi (mean = 0.24, SD = 0.35) were the highest abundance taxa in the non-overweight group (Figure 1, Supplementary Table S1).

### 3.2. Differential Abundance Analysis

We analysed differentially abundant features between study groups using the ANCOM-II tool, which performs pairwise statistical tests between each feature and the target variable. ANCOM-II enhances result reliability through a feature filtering pipeline that eliminates features with a high proportion of zeroes among samples. In comparing AD samples to non-dermatitis samples, ANCOM-II included 19 taxa immediately after birth, 15 taxa at 6 months, and 25 taxa at 18 months after birth. Similarly, when comparing overweight to non-overweight samples, 18 taxa were included immediately after birth, 13 at 6 months, and 26 taxa at 18 months after birth (Supplementary Table S3). ANCOM-II did not find any feature to be differentially abundant between AD and non-AD samples or overweight and normal weight samples at any sampling time (Supplementary Table S3). Additionally, none of the pairwise tests passed at any time point for any feature between AD and non-dermatitis or overweight and non-overweight samples.

### 3.3. Alpha and Beta Diversity

**Figure 2:**
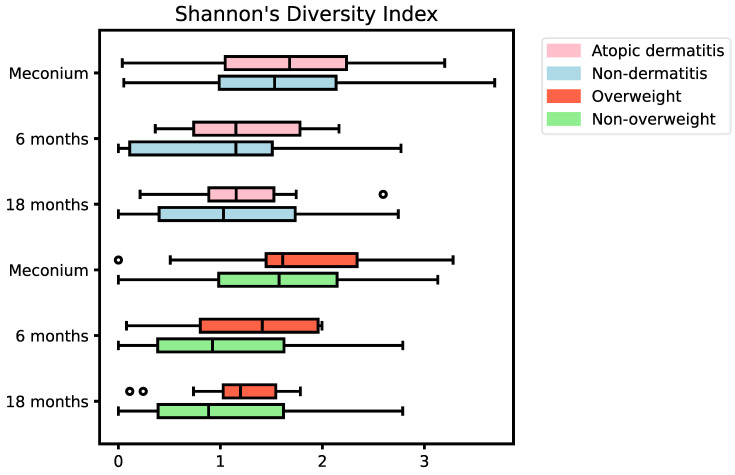
Alpha diversity of gut mycobiome at birth, 6 months, and 18 months according to subsequent AD and overweight. Statistical tests showed no significant differences. Black circles indicate outliers.

Alpha diversity, within sample diversity, showed no significant differences in the Shannon diversity index of the gut mycobiome diversity between AD (mean = 1.61, SD = 0.81) and non-dermatitis (mean = 1.61, SD = 0.95) samples immediately after birth (adjusted *p*-value = 0.75), 6 months after birth (AD, mean = 0.98, SD = 0.75 vs. non-dermatitis, mean = 1.3, SD = 0.61, *p*-value = 0.57), and 18 months after birth (AD, mean = 1.05, SD = 0.78 vs. non-dermatitis, mean = 1.24, SD = 0.61, adjusted *p*-value 0.57) (Figure 2, Supplementary Table S4). Similarly, no significant differences were detected when comparing the gut mycobiome alpha diversity between overweight (mean = 1.52, SD = 0.86) and non-overweight (mean = 1.8, SD = 0.86) samples immediately after birth (adjusted *p*-value = 0.57), 6 months after birth (overweight, mean = 1.03, SD = 0.8, vs. non-overweight, mean = 1.27, SD = 0.73, adjusted *p*-value = 0.57), and 18 months after birth (overweight, mean = 1.03, SD = 0.81, vs. non-overweight, mean = 1.14, SD = 0.51, adjusted *p*-value = 0.57) (Figure 2, Supplementary Table S4).

**Figure 3:**
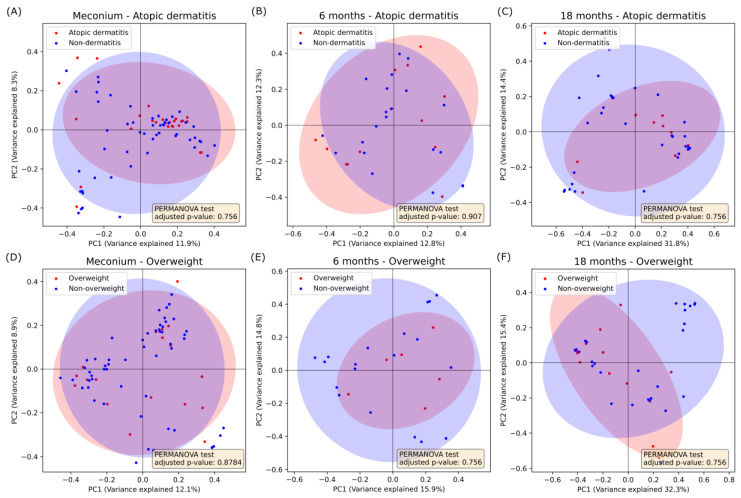
Beta diversity in gut mycobiome samples according to subsequent AD or overweight diagnoses. Gut mycobiome at birth (meconium) (**A**,**D**), 6 months of age (**B**,**E**) and 18 months of age (**C**,**F**) are presented. Beta diversity analyses were performed using the Bray–Curtis dissimilarity based on the diagnosis of AD (**A**–**C**) and overweight (**D**–**F**). *p*-values were adjusted using the Benjamini–Hochberg procedure for multiple testing. No statistically significant differences were found.

When examining the beta diversity, between sample diversity, of gut mycobiome using Bray–Curtis dissimilarity, no significant differences between sample diversity was detected between AD and non-dermatitis samples immediately after birth (PERMANOVA, adjusted *p*-value = 0.76), 6 months after (PERMANOVA, adjusted *p*-value = 0.91), and 18 months after birth (PERMANOVA, adjusted *p*-value = 0.76) (Figure 3, Supplementary Table S5). Similarly, no significant differences were found when comparing overweight and non-overweight samples immediately after birth (PERMANOVA, adjusted *p*-value = 0.88), 6 months after birth (PERMANOVA, adjusted *p*-value = 0.76), and 18 months after birth (PERMANOVA, adjusted *p*-value = 0.76) (Figure 3, Supplementary Table S5).

### 3.4. Machine Learning Classification

**Figure 4:**
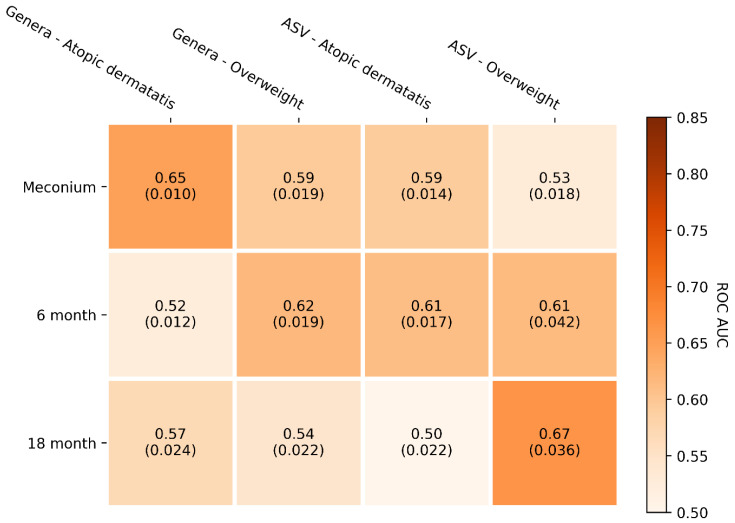
Machine learning model performance in predicting subsequent AD and overweight based on gut mycobiome. Models were trained on mycobiome data in faecal samples obtained at birth (meconium) and 6 months and 18 months of age. The mean AUC values are reported from 40 repeated cross-validation runs. Standard deviation is noted in parentheses below the AUC value.

**Figure 5:**
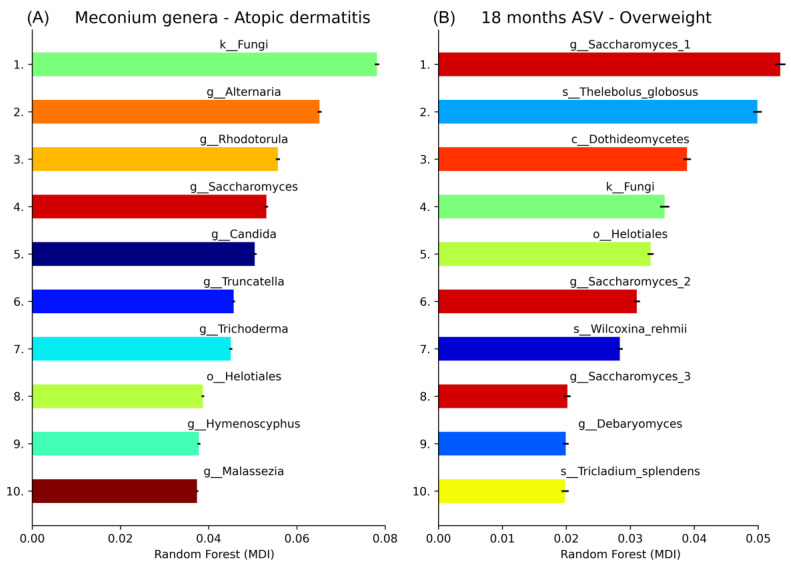
Feature importance from models with the best performance in predicting subsequent diseases in children based on gut mycobiome. (**A**) Subsequent AD using gut mycobiome at birth (meconium samples). (**B**) Overweight using gut mycobiome at 18 months of age. MDI indicates a mean decrease in impurity (MDI) during random forest model training. Black error bars indicate the standard deviation.

The best performing ML models could predict subsequent AD with an AUC of 0.65 (SD = 0.010) using the first stool samples after birth (Figure 4) and overweight with an AUC of 0.67 AUC (SD = 0.036) using the samples obtained at 18 months of age (Figure 4 Supplementary Table S6). Unidentified fungi, *Alternaria*, *Rhodotorula*, *Saccharomyces,* and *Candida* were the highest importance features for the model predicting AD from meconium samples, while taxa identified as *Saccharomyces*, *Thelebolus*, *Dothideomycetes*, Unidentified fungi, and *Helotiales* were the highest importance features when predicting overweight using faecal samples sampled 18 months after birth (Figure 5). Other less important features are visualised (Figure 5), and the exact feature importance values can be found in the supplementary tables (Supplementary Tables S7 and S8).

### 3.5. Analyses of Non-Overlapping Study Groups

**Figure 6:**
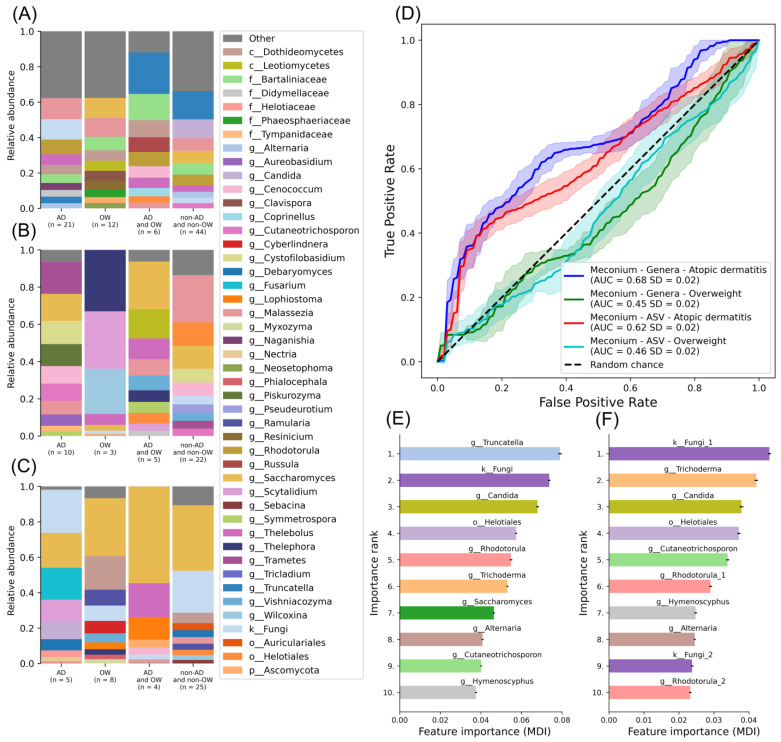
Faecal mycobiota analysis results from non-overlapping study groups. Faecal mycobiota mean relative abundance samples (**A**) at birth, (**B**) 6 months, and (**C**) 18 months of individuals with AD and with normal weight (in figure as “AD”), individuals with overweight but without AD (in figure as “OW”), individuals with AD and overweight (in figure as “AD and OW”), and individuals without AD but with normal weight (in figure as “non-AD and non-OW”) diagnosed at 18 months or 36 months of age. Features are collapsed to the last known taxonomic level down to genus. (**D**) ML model performances of when classifying individuals with AD from individuals without AD or overweight using genera collapsed and ASV relative abundance feature data. (**E**,**F**) Ten most important features are visualised from the two best models with positive error bars visualised as black lines.

Subsequently, the study groups were segregated into non-overlapping categories based on the diagnosis of AD or overweight at 18 or 36 months. Clinical characteristics of these non-overlapping study groups are presented in Supplementary Table S9. The most prevalent feature observed post-birth in individuals with AD and normal weight was *Malassezia* (mean = 0.12, SD = 0.28), while in individuals without AD but with overweight, it was *Saccharomyces* (mean = 0.11, SD = 0.27). In individuals with both AD and overweight, *Truncatella* dominated (mean = 0.23, SD = 0.31), and in those without AD but with normal weight, the most dominant taxon was also *Truncatella* (mean = 0.16, SD = 0.22) (Figure 6A, Supplementary Table S10). At the 6-month sampling point, *Trametes* (mean = 0.17, SD = 0.31) was the most common in individuals with AD and normal weight, *Thelephora* (mean = 0.33, SD = 0.47) in individuals with overweight but without AD, *Saccharomyces* in individuals with both AD and overweight, and *Malassezia* (mean = 0.25, SD = 0.4) in individuals with normal weight but without AD (Figure 6B, Supplementary Table S10). At 18 months after birth, the feature comprising unidentified fungi (mean = 0.24, SD = 0.33) dominated in individuals with AD and normal weight, *Saccharomyces* (mean = 0.33, SD = 0.32) in those with overweight but without AD, *Saccharomyces* (mean = 0.55, SD = 0.32) in individuals with both AD and overweight, and *Saccharomyces* (mean = 0.37, SD = 0.39) in individuals without AD but with normal weight (Figure 6C, Supplementary Table S10). Consistent with the findings from overlapping groups (Figure 1D–F), *Saccharomyces* predominated in all study groups at 18 months of age, except in individuals with AD and normal weight.

Analysis of faecal mycobiota alpha- and beta-diversity post-birth, at 6 months, and at 18 months revealed no significant differences within or between individuals in non-overlapping study groups diagnosed with AD, overweight, or both conditions, compared to those without such diagnoses at 18 or 36 months (Supplementary Tables S11 and S12). Differential abundance analysis of faecal mycobiota across these time points using ANCOM-II revealed no differentially abundant features between individuals with AD, overweight, or both conditions compared to those without such diagnoses at 18 or 36 months (Supplementary Table S13).

Machine learning analyses aimed at predicting individuals with AD and normal weight from those without AD but with normal weight at 18 or 36 months, using faecal mycobiota samples collected post-birth, and exhibited poor predictive performance (AUC = 0.68, SD = 0.02) (Figure 6D). Models trained to predict individuals with overweight but without AD from those without AD or overweight achieved an AUC of 0.62 (SD = 0.02) (Figure 6D). Other models did not surpass the performance of a random chance classifier. Feature importance analysis highlighted *Truncatella*, unidentified fungi, *Candida, Heliotiales, Rhotodorula, Trichoderma*, and *Saccharomyces* among the most important features when predicting AD diagnosed at 18 or 36 months based on faecal mycobiota ASV or genera data sampled post-birth (Figure 6E,F, Supplementary Tables S14 and S15).

## 4. Discussion

In this prospective cohort study, we found no statistically significant associations between early gut mycobiome and subsequent AD or overweight in young children. The ML models showed, however, some capability in predicting later AD with the gut mycobiome at birth and overweight with the gut mycobiome at 18 months of age.

In AD, immune system deregulation, genetic factors, and epidermal dysfunction are some of the leading factors in the pathogenesis of the disease [41]. Only a few studies have previously examined the connection of gut mycobiome to AD in children [26]. A case–control study involving 34 infants of 9 to 12 months of age has reported elevated gut mycobiome alpha diversity relative to both healthy infants and those who had overcome AD [26]. This study also emphasised the significant enrichment of *Rhodotorula* within the gut mycobiome of infants with ongoing AD [26]. Interestingly, our results show that *Rhodotorula* was an important feature when predicting later AD using meconium samples. Furthermore, a case–control study of 97 children identified differences in the metabolome of faecal mycobiome that were associated with subsequent atopic wheeze diagnoses [29]. We did not investigate the metabolome of gut mycobiome in the present study.

In addition to gut mycobiome, skin bacterial and fungal microbiome dysbiosis have been associated with AD [41,42]. The relative abundance of *Malassezia globosa* has been reported to be decreased in the skin of AD patients, while the relative abundance of *Malassezia dermatitis* and *Malassezia symbodialis* have been increased [43]. Some species of *Malassezia* [44], *Candida* [45], and *Cryptococcus* [45] have been found exclusively from AD skin. Studies investigating the relation of AD to the gut mycobiome have been scarce, both in children and in adults. In our study, we found that *Malessezia* and *Candida* were the tenth and fifth most important genera when predicting later AD with gut mycobiome at birth.

Several factors contribute to the pathogenesis of obesity, such as diet, lifestyle, gut microbiome, and genetic factors [46]. Gut bacteriome has been extensively researched in relation to obesity [47]. Gut bacteria have been shown to produce short-chain fatty acids, tryptophan metabolites, and lipopolysaccharides, which have roles in regulating immunity, inflammation, metabolism, and appetite [47]. Bacteria have been shown to change the activity and availability of bile acids, which facilitate dietary fat and fat-soluble vitamin absorption in the intestinal lumen [47,48]. Similarly, several studies suggest that gut mycobiome dysbiosis might be associated with obesity [49]. The pathogenesis of obesity is likely a complex network of interactions between the host, bacteria, and fungi [50]. Abundances of the genera *Candida* and *Aspergillus* have been found to be higher in the obese group in adults, while fungi from genus *Mucor* have been reported to be depleted in the non-obese group [19]. Additionally, one study has found that the abundances of fungi from the genera *Alternaria, Saccharomyces, Tilletiopsis*, and *Septoriella* were reduced in obese mice [51]. A previous case–control study found that fungi from *Candida* and *Rhodotorula* were enriched in the obese group in adults [52]. Decreased mycobiome diversity has been previously associated with obesity in adults [19]. There are limited data on the gut mycobiome in children with obesity. In the present study, several fungi classified as *Saccharomyces* were important when predicting overweight according to ML models. 

The present study was a prospective cohort study which collected samples at birth and at age of 6 and 18 months and followed up children until the age of 3 years. There are few studies examining mycobiome in paediatric and adolescent populations. A case–control study encompassing 93 children aged 3.25 to 19 years revealed an enrichment of *Saccharomyces cerevisiae* within the gastrointestinal tracts of those afflicted with Crohn’s disease compared with both patients with ulcerative colitis and healthy counterparts [23]. We found that several fungi classified as *Saccharomyces* were important when predicting overweight using the gut mycobiome. Another case–control study focused on 124 paediatric patients aged 2 to 18 years, identifying a heightened prevalence of *Candida tropicalis* in those diagnosed with Crohn’s disease, as opposed to their healthy peers [24].

Our study has several strengths. In the present study, we used a prospective study design, and the samples were gathered before clinical outcomes occurred. We followed up the children from birth up to the age of 3 years. Faecal samples collected at birth, 6 months after birth, and 18 months after birth allowed the analyses on later AD and overweight diagnoses. We had high-quality growth data based on standardised growth measurements for analyses of overweight. One significant strength of the present study was our utilisation of 50 negative controls during DNA extraction and seven in the PCR process, enabling the removal of contaminant sequences from the dataset. Additionally, we deployed a balanced set of analyses to investigate the association of gut mycobiome composition with AD and overweight.

Our study has its limitations. We could not account for confounding variables in the machine learning analyses, potentially limiting the model’s ability to generalise to unseen samples. Another limitation is the low amount of PCR negative controls; a higher number would allow for more accurate contamination removal. Additionally, very rare sequences were filtered out, which might be important when analysing groupwise differences between study groups. Based on our results, the role of mycobiome in relation to these diseases is complex and is most likely affected by gut bacteria and other factors, while we only investigated the role of mycobiome alone. Another limitation is that we could not study obese or morbidly obese children, as our cohort, after processing samples, only had a sufficient number of overweight individuals remaining for analysis. Furthermore, there are several factors and mechanisms that might contribute to the pathogenesis of obesity, which we were not able to take into account in the present study. A significant limitation in our study’s investigation of AD is our inability to differentiate between different types of AD, as we solely rely on physician-diagnosed AD data. In the present study, we investigated the composition of faecal mycobiota. However, it is important to note that this analysis represents the mycobiota of the distal intestines rather than the intestines as a whole. Sample sizes were limited in groups with faecal samples sampled at 6 months and 18 months of age for AD, allowing only for a simple ML model building scheme. Additionally, we did not have an outside validation set of data to validate our ML model performance.

## 5. Conclusions

The present study investigated the association between mycobiome and non-communicable diseases in a prospective paediatric cohort study. Here, we found a weak association between early gut mycobiome composition and subsequent overweight and AD when using ML models. Yet, the role of mycobiome in these diseases is likely a part of the complex network of bacterial and fungal interactions.

## Figures and Tables

**Table 1:** Faecal samples according to sampling time and clinical outcomes. Some samples were used to investigate both AD and overweight. In the initial (“overlapping study groups”) analyses, a total of 194 unique samples are included.

	Number of Samples			
Faecal sampling time	AD	Non-dermatitis	Overweight	Non-overweight
Overlapping groups				
First stool after birth	29	66	19	67
6 months	16	33	8	33
18 months	10	35	13	30
	Only AD	Only overweight	AD and overweight	Non-AD and normal weight
Non-overlapping groups				
First stool after birth	21	12	6	44
6 months	10	3	5	22
18 months	5	8	4	25

## Data Availability

Sequence data were uploaded to BioProject with the accession number PRJNA831656.

## Supplementary Materials

The following supporting information can be downloaded at: https://www.mdpi.com/article/10.3390/jof10050333/s1, Table S1: Clinical characteristics of study groups, Table S2: Average relative abundances of taxonomic features collapsed to the last identified level down to genus, Table S3: Differential abundance ANCOM-II raw output for each group, Table S4: Alpha diversity results with Kruskal–Wallis H test for significant differences between study groups, Table S5: Beta diversity PERMANOVA results using Bray–Curtis dissimilarity, Table S6: ROC and PR performances trained on the data and dummy classifiers representing random chance, Table S7: Feature importances of the best models in predicting AD and overweight, Table S8: Taxonomic classification results of ASVs with confidence values, Table S9: Clinical characteristics of non-overlapping study groups, Table S10: Average relative abundances of taxonomic features collapsed to the last identified level down to genus using non-overlapping study groups, Table S11: Beta diversity analysis results of non-overlapping study groups, Table S12: Alpha diversity analysis results of non-overlapping study groups, Table S13: Differential abundance analysis results of non-overlapping study groups, Table S14: Feature importance results of non-overlapping study groups, Table S15: Taxonomic classification information of each feature in the ASV data of non-overlapping data in the meconium sampling point.
